# QIAD assay for quantitating a compound’s efficacy in elimination of toxic Aβ oligomers

**DOI:** 10.1038/srep13222

**Published:** 2015-09-23

**Authors:** Oleksandr Brener, Tina Dunkelmann, Lothar Gremer, Thomas van Groen, Ewa A. Mirecka, Inga Kadish, Antje Willuweit, Janine Kutzsche, Dagmar Jürgens, Stephan Rudolph, Markus Tusche, Patrick Bongen, Jörg Pietruszka, Filipp Oesterhelt, Karl-Josef Langen, Hans-Ulrich Demuth, Arnold Janssen, Wolfgang Hoyer, Susanne A. Funke, Luitgard Nagel-Steger, Dieter Willbold

**Affiliations:** 1 Institute of Complex Systems, Structural Biochemistry (ICS-6), Research Centre Jülich, 52425 Jülich, Germany; 2 Institut für Physikalische Biologie, Heinrich-Heine-Universität Düsseldorf, 40225 Düsseldorf, Germany; 3 Department of Cell, Developmental, and Integrative Biology, University of Alabama at Birmingham, Birmingham, AL, USA; 4 Institute of Neuroscience and Medicine (INM-4), Research Centre Jülich (FZJ), 52425 Jülich, Germany; 5 Institute for Bioorganic Chemistry, Heinrich-Heine-Universität Düsseldorf, 52426 Jülich, Germany; 6 Institut für Bio- und Geowissenschaften: Biotechnologie (IBG-1), Forschungszentrum Jülich, 52428 Jülich, Germany; 7 Fraunhofer Institute for Cell Therapy and Immunology, Dep. Molecular Drug Biochemistry and Therapy, 06120 Halle, Germany; 8 Bioanalytik, Hochschule für Angewandte Wissenschaften, Coburg, Germany; 9 Institute of Mathematics, Lehrstuhl für Statistik und Wahrscheinlichkeitstheorie, Heinrich-Heine-Universität Düsseldorf, 40225 Düsseldorf, Germany

## Abstract

Strong evidence exists for a central role of amyloid β-protein (Aβ) oligomers in the pathogenesis of Alzheimer’s disease. We have developed a fast, reliable and robust *in vitro* assay, termed QIAD, to quantify the effect of any compound on the Aβ aggregate size distribution. Applying QIAD, we studied the effect of homotaurine, scyllo-inositol, EGCG, the benzofuran derivative KMS88009, ZAβ3W, the D-enantiomeric peptide D3 and its tandem version D3D3 on Aβ aggregation. The predictive power of the assay for *in vivo* efficacy is demonstrated by comparing the oligomer elimination efficiency of D3 and D3D3 with their treatment effects in animal models of Alzheimer´s disease.

Alzheimer’s disease (AD) is a progressive neurodegenerative disorder, which is the most common cause of dementia. Growing evidence exists that instead of amyloid β-protein (Aβ) monomers or fibrils, small and diffusible Aβ oligomers seem to be decisive for disease development and progression^1^. Thus, the potency to eliminate Aβ oligomers is one of the most desirable criteria for the selection of agents as lead compounds for drug development towards AD treatment^2^. Any screening for oligomer eliminating compounds requires a well characterized target. Therefore, new methods for the preparation, purification and quantification of specific Aβ oligomers, which are representative for the toxic oligomers involved in AD pathogenesis, are urgently needed in AD drug development. Quantitative assessment of Aβ assembly size-distributions is difficult, because the heterogeneity of *in vitro* obtained Aβ assemblies^3^ impedes most of the standard analytical methods.

We have designed an assay for the quantitative determination of interference with Aβ aggregate size distribution (QIAD). The QIAD assay, based on a combination of density gradient ultracentrifugation (DGC) and reversed phase high performance liquid chromatography (RP-HPLC), permits the quantitative analysis of the impact of any compound on Aβ aggregation. We applied QIAD on several compounds, i.e., homotaurine, scyllo-inositol, EGCG, the benzofuran derivative KMS88009, ZAβ3W, the D-enantiomeric peptide D3, and its tandem version D3D3. Comparison of QIAD data obtained for D3 and D3D3 with the treatment effects in AD animal models demonstrates the predictive power of the assay for *in vivo* efficacy.

## Results and Discussion

### The principle of the QIAD assay

**Figure 1:**
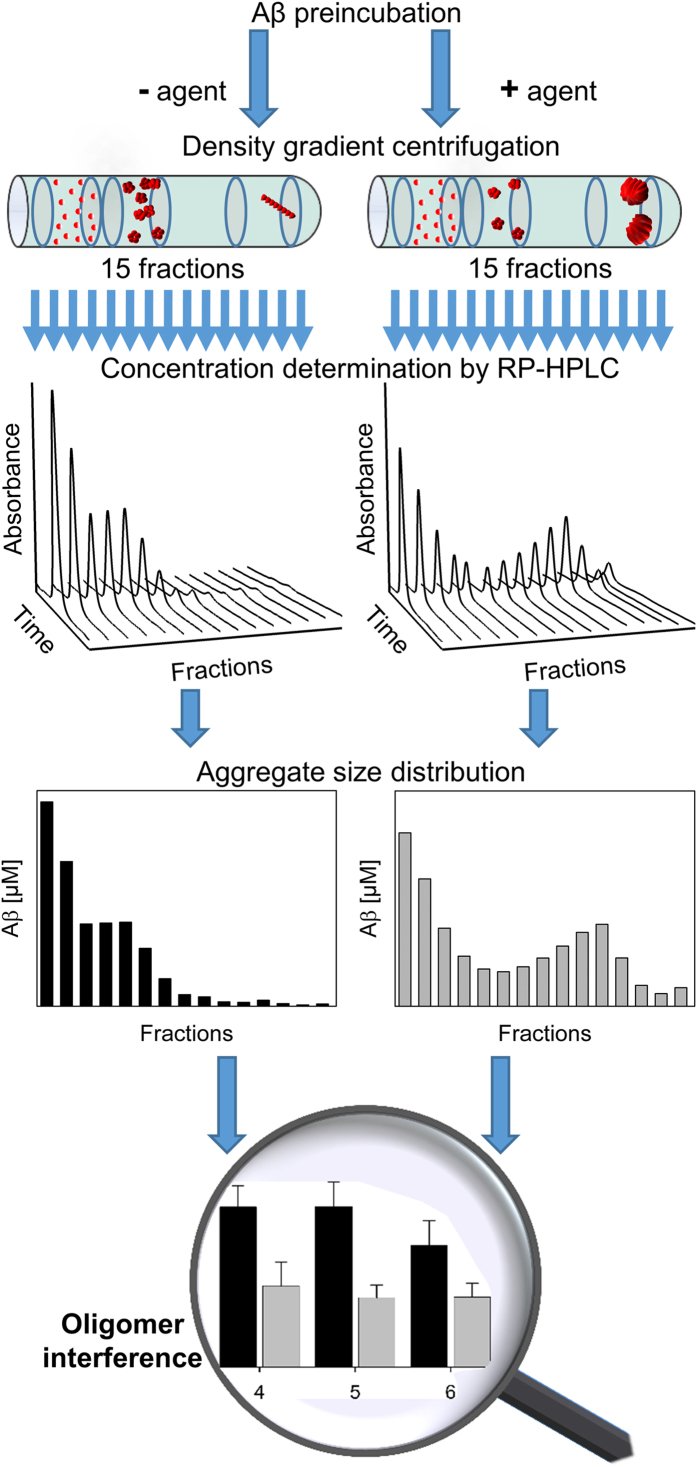
Scheme of the QIAD assay consisting of the following steps i) preparation of Aβ(1–42) assemblies containing the target aggregate species by Aβ(1–42) pre-incubation; ii) incubation with and without the agent of interest; iii) separation of Aβ(1–42) assemblies by density gradient centrifugation and subsequent fractionation; iv) determination of the total Aβ(1–42) amount in each fraction by integrating the Aβ(1–42) absorption signal during RP-HPLC analysis and the concluding visualisation of the Aβ(1–42) oligomer removal. The drawing of the magnifying glass was drawn by O. Brener

**Figure 2:**
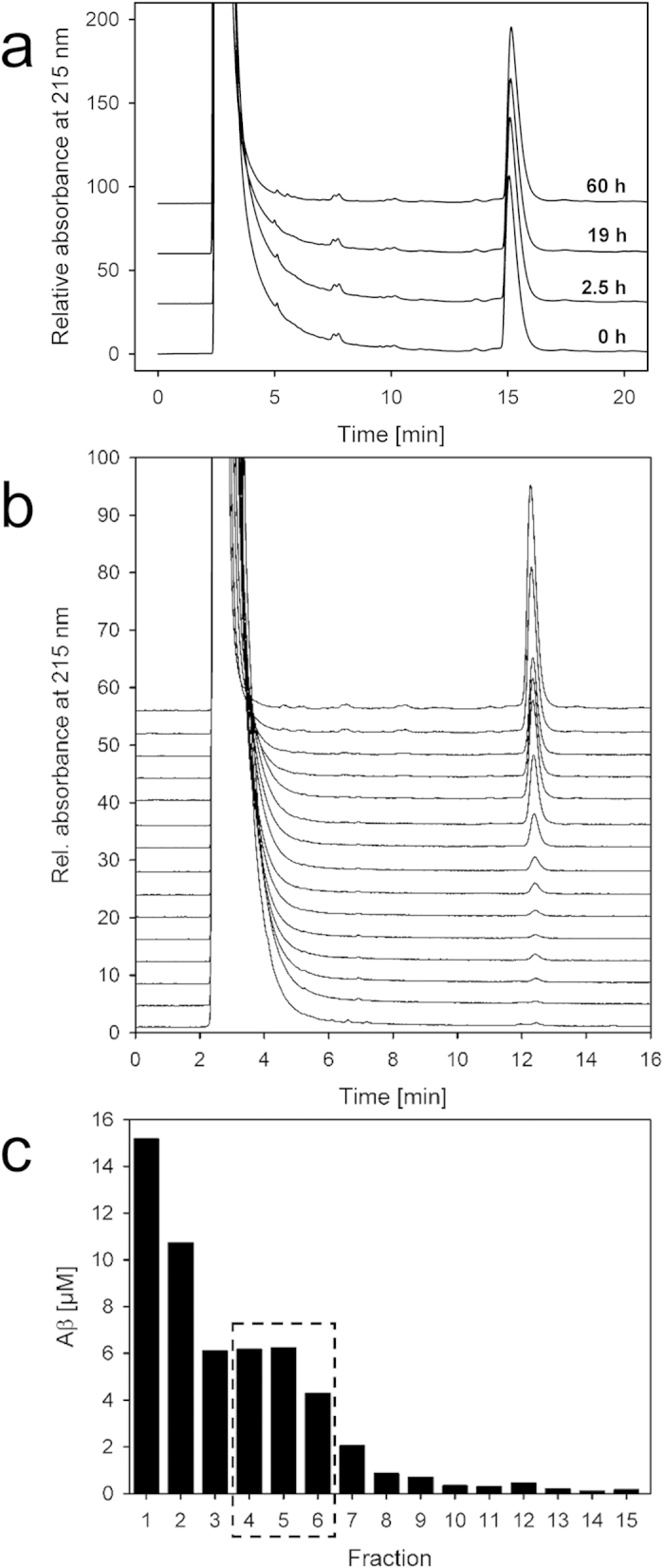
Quantification of Aβ(1–42) in different assembly states by QIAD. **(a)** RP-HPLC chromatograms of 1.8 ng of either freshly resolved or incubated (2.5, 19 and 60 h) Aβ(1–42) were mixed with 40% iodixanol to simulate the properties of the density gradient fractions and analysed by RP-HPLC. Iodixanol elutes clearly separated from Aβ(1–42) (retention time 2-5 min), enabling UV spectroscopic analysis of Aβ(1–42) concentrations. Chromatograms of all Aβ(1–42) samples regardless of their degree of aggregation reveal the same retention times and peak areas. **(b)** Chromatograms for each fraction obtained from density gradient centrifugation (DGC) are presented with a constant offset for clarity. The peak at 2 to 4 min corresponds to the density gradient material iodixanol, whereas the peak at 12.5 min corresponds to Aβ(1-42). **(c)** Bar chart showing the Aβ(1–42) distribution after DGC of pre-incubated Aβ(1–42). Aβ(1–42) concentrations in each fraction were determined by integration of the Aβ(1–42) absorption in the RP-HPLC chromatograms shown in **(b)**. Aβ(1-42) oligomers of interest (dashed box) are located in fractions 4 to 6.

The QIAD assay consists of the following steps (Fig. 1): i) preparation of Aβ(1-42) assemblies containing the target aggregate species by Aβ(1-42) pre-incubation; ii) incubation with and without the agent of interest; iii) separation of Aβ(1–42) assemblies by DGC and subsequent fractionation; and iv) determination of the total Aβ(1–42) amount in each fraction, e.g. by integrating the Aβ(1–42) absorption signal during RP-HPLC analysis. The essential requirement for the quantitative analysis of different Aβ(1–42) aggregates is total disassembly of all different Aβ(1–42) assemblies. We were able to achieve this by the harsh conditions during the RP-HPLC runs (presence of acetonitrile and column heating to 80 °C) converting all Aβ assemblies into a single species with the same retention time. Thus, the integrated peak area of this peak correlates with total Aβ(1–42) amount (Fig. 2a). The obtained results indicate that under these conditions the integrated peak areas were independent from Aβ(1–42) incubation time and thus independent from its aggregation state. Prior to RP-HPLC DGC allows matrix-free separation and fractionation of different Aβ(1–42) assemblies according to their sedimentation coefficients, which are dependent on particle size and shape^4^,^5^. Alternatively the Aβ(1–42) aggregate size distribution could have been studied by a combination of SEC (Size Exclusion Chromatography) and MALS (Multi-Angle Light Scattering) detection. Such approach seems to be more convenient since it would take less time for a single sample and the direct outcome would be a distribution of molecular masses instead of sedimentation coefficients. Nevertheless, DGC was superior to SEC/MALS with regard to the recovery rates of Aβ(1–42). Possible interactions of Aβ(1–42) with the column matrix and the necessity of either an online filtration or centrifugation step prior to chromatography might be responsible for material losses. Aβ(1–42) fibrils, huge aggregates or huge complexes (Aβ/ligand) could not pass through in the AD research field prevalent SEC columns with a void volume of 75 or 200 kDa. For DGC the whole sample can be loaded onto the gradient regardless of the aggregation state of the sample. Additionally, the fact, that for each sample a fresh gradient is prepared, while SEC columns have to be reused, contributes clearly to the very good reproducibility of the analysis. We used steps (i) to (iii) previously to investigate the effect of agent candidates on size distribution of Aβ(1–42) aggregates formed *in vitro*, although only in a qualitative or semi-quantitative way^6^. Here, for the first time, we add the fully quantitative analysis of a compound’s impact on the Aβ(1–42) aggregate size distribution and call it QIAD assay (Fig. 2b,c). The principle of the QIAD assay can easily be transferred to measure the quantitative interference on aggregates consisting of aggregation-prone peptides or proteins other than Aβ. Thus, to avoid any confusion with QIAD variants yet to come, we have termed the Aβ-specific QIAD assay “Aβ-QIAD”.

The method abstains from monomeric initial conditions, instead it starts from a reproducibly adjustable assembly state, where Aβ(1–42) oligomers are enriched by a pre-incubation step. While Aβ recovery rates are rarely demonstrated^7^ in the AD research field, the herein described procedure reliably yielded total Aβ(1–42) recovery rates close to 100%, i.e., 95.6 ± 7.9% for control DGC runs (Aβ without agent) and 89.2 ± 16.1% for DGC runs in the presence of D3 or D3D3. Moreover, the method described here allows the quantification of yields for any DGC-separated Aβ(1–42) fraction within the sample. For example, the average amount of the Aβ(1–42) oligomers (fractions 4 to 6) in eleven independent preparations was 31.7 ± 3.9%.

### Characterisation of Aβ oligomers

**Figure 3:**
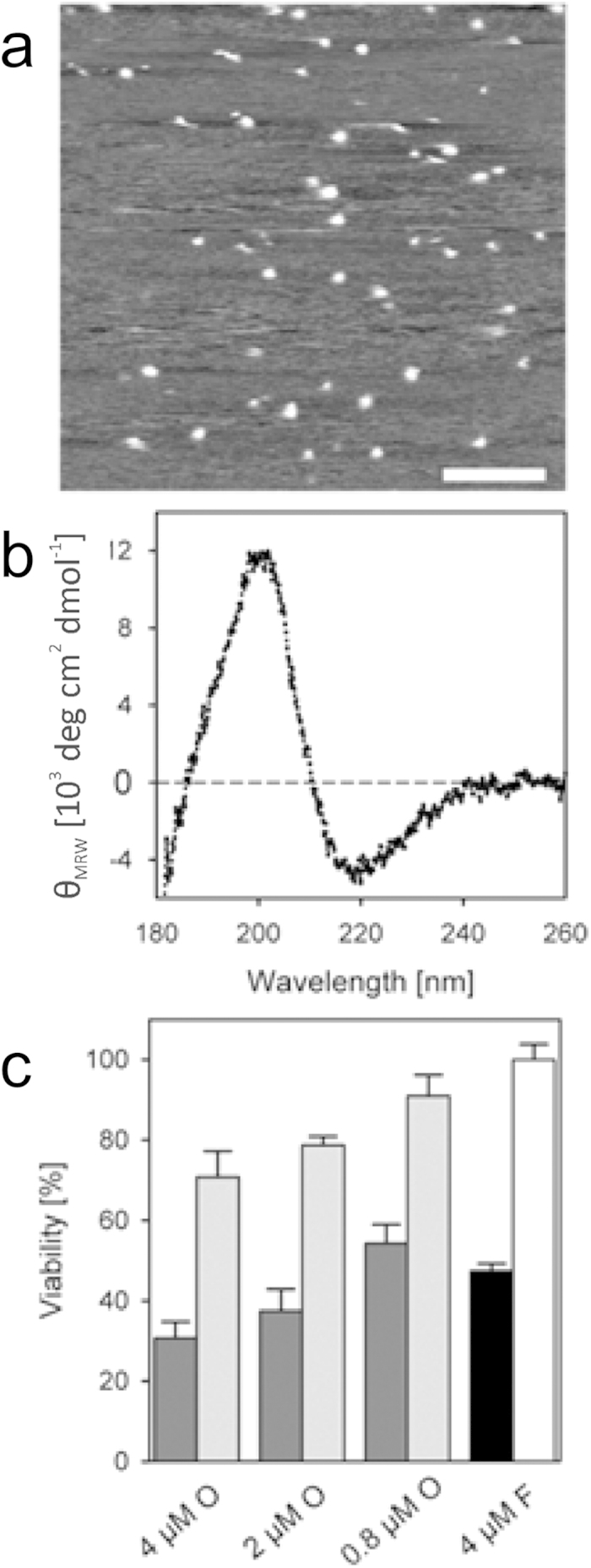
Characterization of Aβ oligomers. **(a)** AFM of Aβ(1–42) oligomers from pooled fractions 5 and 6. Scale bar 200 nm. **(b)** Far-UV CD spectroscopy of Aβ(1–42) oligomers from pooled fractions 5 and 6 indicating a pre-dominantly β-sheet secondary structure. **(c)** Toxicity of Aβ(1–42) oligomers from pooled fractions 5 and 6 tested by MTT cell viability. RA/BDNF differentiated SH-SY5Y cells were treated with Aβ(1–42) oligomers (dark grey) at monomer concentrations of 4, 2 and 0.8 μM with corresponding buffer dilution controls containing 16, 8 and 3.2% iodixanol (light grey), or with ultrasonicated Aβ(1–42) fibrils (black) at a monomer concentration of 4 μM and the corresponding buffer without iodixanol (white) as control. All given Aβ(1–42) assembly concentrations relate to Aβ(1–42) monomers.

Aβ(1–42) assemblies located in fractions 4 to 6 were specifically sensitive to our agents D3^6^,^8^,^9^,^10^ and D3D3. Therefore, these species were further characterized in detail. According to the calibration of the density gradient with a set of globular proteins with known *s*-values, the Aβ(1–42) assemblies in fractions 4 to 6 had *s*-values of about 7 S (Supplementary Figure 1a). Atomic force microscopy (AFM) analysis of fraction 4 to 6 revealed assemblies in spherically shaped particles with heights of 4.7 nm and diameters corrected for the tip dimensions of 8.7 nm (Fig. 3a and Supplementary Figure 2). Modelled as oblate spheroids with an axial ratio of ~2, these Aβ(1–42) oligomers have a molecular weight of about 104 kDa, which corresponds to about 23 monomeric units. This value is in good accordance with the estimated *s*-value of 7 S. Circular dichroism (CD) measurements of the oligomers revealed a spectrum with β-sheet characteristics (Fig. 3b). However, since the Aβ(1–42) particles were negative for Thioflavin T (ThT) fluorescence staining (Supplementary Figure 3), we conclude that the secondary structure of the oligomers differs from regular amyloid cross-β fibrils, which typically exhibit strong ThT fluorescence. The DGC derived Aβ(1–42) oligomers exhibited high cytotoxicity to differentiated human SH-SY5Y neuroblastoma cells^11^,^12^,^13^ (Fig. 3c), thus meeting a further criterion of toxic Aβ(1–42) oligomers involved in AD pathogenesis.

### Aβ oligomer elimination by D3 and D3D3

**Figure 4:**
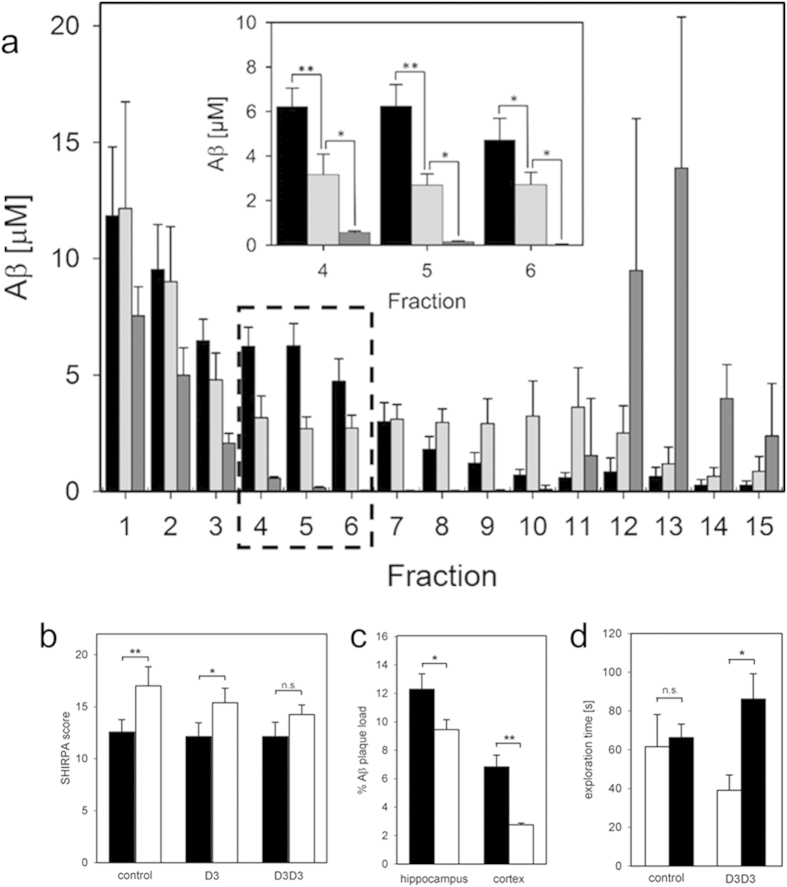
Effects of D3 and D3D3 *in vitro* and *in vivo*. **(a)** QIAD assay. After pre-incubation of 80 μM Aβ(1–42) for 4.5 h at RT and further incubation for 40 min at RT with or without agent, Aβ(1–42) size distributions in the absence (black) or presence of either D3 (light gray, 32 μg/ml, 20 μM) or D3D3 (dark gray, 32 μg/ml, 10 μM) were analyzed by DGC. Aβ(1–42) concentrations as determined by UV absorption during RP-HPLC are shown with standard deviations. Aβ(1–42) oligomers of interest (dashed box) are located in fractions 4 to 6. (Inset) Magnification of boxed area displaying significant differences of Aβ(1–42) oligomer contents between untreated (black), D3 treated (light grey) and D3D3 treated Aβ(1–42) (dark grey). Paired U-test; *p ≤ 0.05, **p ≤ 0.01 **(b)** Assessment of TBA 2.1 phenotype using part of the SHIRPA test battery before (black) and after i.p. treatment (white) for 4 weeks with phosphate buffered saline (n = 7), or D3 (n = 8) or D3D3 (n = 8) (5.1 ± 0.1 mg per kg body weight per day). Repeated measures ANOVA before vs. after p = 0.0027, paired t-test (hypothesized difference ≥0) before vs. after, *p = 0.035, **p = 0.010. **(c)** Plaque load reduction of D3D3 treated Tg-SwDI mice (n = 10) (white) as compared to saline-treated controls (n = 9) (black) in hippocampus and cortex. Unpaired t-test (hypothesized difference ≥0) D3D3 vs. saline, *p = 0.020, **p < 0.0001. **(d)** Object recognition test of Tg-SwDI mice treated either with D3D3 or saline (control). Preference for the new object (black) is expressed as exploration time in comparison with exploration of a familiar object (white); Paired t-test (hypothesized difference ≥0) before vs. after, **p = 0.016. n.s.: not significant (p > 0.05).

Here, we demonstrate the power of the Aβ-QIAD assay by quantitatively comparing the outcome of the assay for two therapeutically interesting agents. The first agent is a mirror image phage display^8^,^14^ selected D-enantiomeric peptide, termed “D3”, which inhibits the formation of regular Aβ(1–42) fibrils, removes Aβ(1–42) oligomers and reduces Aβ(1–42) cytotoxicity *in vitro*. *In vivo*, D3 binds to amyloid plaques^10^, is able to reduce plaque load, decrease inflammation and enhance cognition in a transgenic AD mouse model even after oral application^6^,^9^. The second agent is a head-to-tail tandem version of D3, termed “D3D3”, that is expected to have enhanced efficacy due to increased avidity. As shown in Fig. 4a, both agents yielded significant reduction of Aβ(1–42) oligomers in DGC fractions 4 to 6 in comparison to agent-free controls. D3 (32 μg/ml, 20 μM) and D3D3 (32 μg/ml, 10 μM) reduced Aβ(1–42) oligomers by 50% and 96%, respectively. The oligomer elimination efficiency is defined as the percentage of reduction of Aβ(1–42) contents in fractions 4 to 6 in presence of agent as compared to absence of agent. D3D3 proved to be significantly more efficient than D3 (Fig. 4a and Supplementary Figure 4). Notably, monomeric Aβ(1–42) located in DGC fractions 1 and 2 were negligibly affected by D3 and D3D3. The net reduction of Aβ(1–42) oligomers by D3 and D3D3 was balanced by an increase of high-molecular weight aggregates located in DGC fractions 9 to 12 or in fractions 12 to 15, respectively (Fig. 4a and Supplementary Figure 4). Increase of Aβ(1–42) content in DGC fractions 9 to 12 or in fractions 12 to 15 does not mean that formation of pre-fibrils or fibrils is induced by D3 and D3D3. The Aβ(1–42) species in fractions 9 to 15 have completely different morphologies depending on presence or absence of D3 or D3D3. Thus, D3 and D3D3 are active *in vivo* and obviously both have the same mechanism of action, which is elimination of Aβ(1–42) oligomers of the same size as shown *in vitro* in the QIAD assay, with D3D3 being more efficient than D3, and with D3D3 yielding larger co-aggregates than D3. D3-Aβ-co-aggregates were shown previously to be non-toxic, non-amyloidogenic, amorphous and ThT negative^6^.

### Correlation of the *in vitro* and *in vivo* results

To verify the hypothesis that increased efficiency in Aβ oligomer elimination of an agent correlates with *in vivo* potency of the agent, therapeutic effects of D3 and D3D3 were investigated in the transgenic mouse model TBA2.1, which is expressing human N-terminally truncated and pyroglutamated Aβ^15^. Thus, the analysis of protein levels in brains of TBA2.1 mice showed substantial amounts of both Aβ and pE3–Aβ^15^. Three groups of TBA2.1 mice were treated either with phosphate buffered saline, D3 or D3D3 (5.1 ± 0.1 mg per kg body weight per day, i.p., for 4 weeks using minipumps). Their phenotypes before and after the treatment were assessed using SHIRPA tests^16^. Importantly, D3D3-treated mice did not show significant worsening of their phenotypes in the SHIRPA test, whereas the phenotypes of D3-treated mice and saline-treated mice worsened significantly, during the four weeks duration of the experiment (Fig. 4b). Thus, D3D3 was more efficient than D3, exactly as predicted from the Aβ-QIAD assay results of both compounds. Despite this nice correlation, the observed *in vivo* effects of D3 and D3D3 treatment are not necessarily exclusively due to the Aβ oligomer elimination activity of both compounds. One may well think of other potential activities, e.g. interference with described Aβ oligomer receptors like cellular prion protein^17^,^18^,^19^, N-methyl-D-aspartate receptor or paired immunoglobulin-like receptor B^20^, overall inflammation reduction^9^, or interference with the postulated Aβ oligomer induced cell membrane permeation^1^,^21^,^22^.

After having compared efficacies of D3 and D3D3 in the TBA2.1 model, we wanted to further verify the *in vivo* efficacy of D3D3, and thus, treated the transgenic AD mouse model Tg-SwDI^23^ with this peptide. Intraperitoneal application of D3D3 (1.4 ± 0.15 mg per kg body weight per day for 4 weeks, using implanted minipumps) led to significant reduction of plaque load in comparison to the saline treated control group (Fig. 4c and Supplementary Figure 5). Cognitive deficits of Tg-SwDI mice, as measured in the object recognition test (Supplementary Figure 6), were significantly improved upon D3D3 treatment (Fig. 4d). Behavioural side effects caused by D3D3 treatment as measured by open field and zero maze tests were not detected.

### Further compounds tested by QIAD

**Figure 5:**
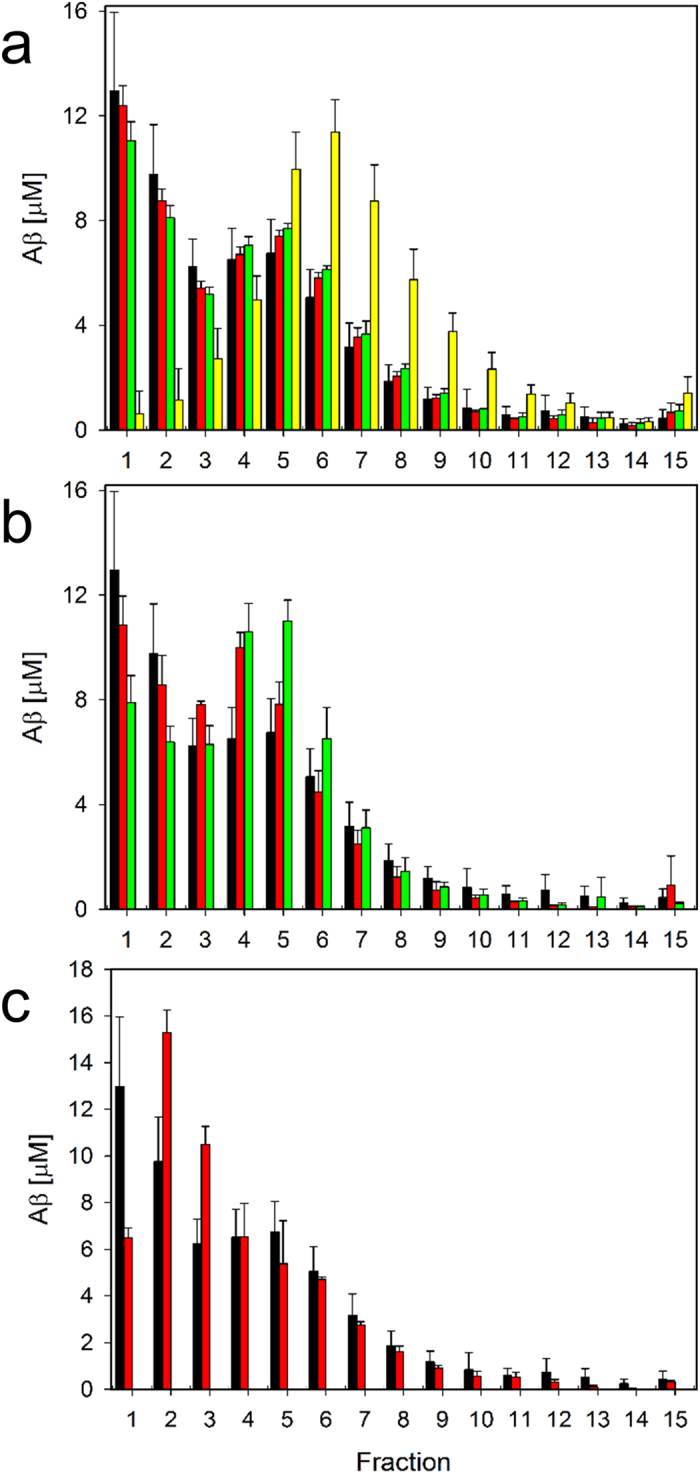
QIAD assay results of agents that were previously reported to influence Aβ(1–42) assembly distribution and/or did show efficacy in cognition improvement of transgenic AD animals. Shown are Aβ(1-42) particle size distributions after pre-incubation of 80 μM Aβ(1-42) for 4.5 h at RT and further incubation for 40 min without (black bars) or with agent. Agents were **(a)** 2 mM homotaurine (red), scyllo-inositol (green) or epigallocatechin gallate (yellow), **(b)** 200 μM 6-methoxy-2-(4-dimethylaminostyryl) benzofuran (KMS88009, green), and **(c)** 54 μM ZAβ3W (red). Note that the assay with KMS88009 contained 1.6% DMSO and 0.2% Tween20 (final concentrations). Therefore, the control without agent was done in presence of 1.6% DMSO and 0.2% Tween20 (red bars in **(b)**).

To demonstrate the usefulness of the QIAD assay, we applied it to a number of agents, which were previously reported to influence Aβ(1–42) assembly distribution and/or did show efficacy in cognition improvement of transgenic AD animals, like homotaurine (Alzhemed), scyllo-inositol, and epigallocatechin gallate (EGCG). EGCG clearly converted Aβ(1–42) monomers into larger particles (Fig. 5a and Supplementary Figure 7), exactly as had been reported^24^. Homotaurine and scyllo-inositol did not have any significant effect on the Aβ(1–42) aggregate size distribution even at 2 mM concentrations (Fig. 5a). This is in agreement with recent work reporting that scyllo-inositol does not have any effect on Aβ toxicity and Aβ oligomer formation^25^. Also, for homotaurine negative results on Aβ aggregation were reported and discussed in relation to failed clinical trials^26^.

Recently, 6-methoxy-2-(4-dimethylaminostyryl) benzofuran (KMS88009) (Supplementary Figure 8) was reported to reduce oligomeric Aβ species and to reverse cognitive deficits in AD transgenic mice^27^,^28^. Because KMS88009 was not water soluble, but needed DMSO and Tween20 for solvation, it was necessary to adapt the QIAD assay conditions to contain 1.6% DMSO and 0.2% Tween20 in the solvent (final concentrations). Figure 5b shows the Aβ(1–42) aggregate size distributions with and without KMS88009. This clearly shows that QIAD assay conditions can be adapted to experimental needs, easily. Similar to EGCG, KMS88009 reduces the amount of Aβ(1–42) monomers in DGC fractions 1 and 2 in favour of oligomer sized particles in fractions 5 and 6 (Fig. 5b). This, however, does not necessarily mean that KMS88009 generates toxic Aβ(1–42) oligomers. The increase of Aβ amounts in fractions 4 to 6 is not the opposite of the reduction of Aβ amounts in fraction 4 to 6, because the newly generated particles are most likely not consisting of pure Aβ(1–42), but rather are co-assemblies of Aβ(1–42) and KMS88009.

Another example compound, ZAβ3W, which is an Affibody protein that prevents Aβ toxicity by sequestration of monomeric Aβ^29^,^30^, induced reduction of Aβ(1–42) content in fraction 1 and an increase in Aβ(1–42) content in fraction 2, exactly as expected for the formation of a 1:1 ZAβ3W-Aβ complex with an *s*-value of 2.4 S (Fig. 5c).

### Predictive power of the QIAD assay

The hereby described Aβ-QIAD assay for analysis of agent-induced Aβ oligomer elimination allows comprehensive and reliable *in vitro* screening of drug candidates for maximum efficacy in the elimination of cytotoxic Aβ oligomers. Analysis of Aβ(1–42) content in DGC fractions by RP-HPLC allows quantitative comparison between various drug candidates. Although Aβ oligomer elimination is not necessarily the only mechanism of compounds that show *in vivo* efficacy in AD animal models, Aβ oligomer elimination is — from the current point of view — the most promising option for causative AD treatment and therapy. The observed relation between Aβ oligomer elimination of D3 and D3D3 and their *in vivo* results strengthens the role of Aβ oligomers in AD pathology and the role of D3 and D3D3 as potential causative agents for AD treatment. Furthermore, the assay will help to avoid testing of ineffective compounds in animals and time consuming *in vivo* assays or even clinical trials. The QIAD assay is adjustable for any aggregation-prone peptide or protein.

## Methods

### Ethics statement

All experiments were carried out in compliance with the USPHS Guide for Care and Use of Laboratory Animals and approved by the Institutional Animal Care and Use Committee (IACUC) as well as in conformance with the German Protection of Animals Act (TierSchG §§ 7–9) and with permit of an Animal Protection Committee at the local agency (Landesamt für Natur und Verbraucherschutz (LANUV), Northrhine-Westphalia, Germany, permit ID: AZ84-02.04.2011.A359)

### QIAD assay

The QIAD assay involves four steps: (i) pre-incubation of Aβ(1–42); (ii) addition of the agent to oligomer-enriched Aβ(1–42) samples; (iii) the density gradient centrifugation step; and (iv) the quantification of Aβ(1–42) assemblies by RP-HPLC (Fig. 1).

1. *Pre-incubation of Aβ.* Pre-incubation of the Aβ(1–42) solution leads to an increase of the amount of material with higher *s*-values, penetrating deeper into the gradient during centrifugation (details regarding the gradient are given below). A pre-incubation of 80 μM Aβ(1–42) between 4.5 h and 6 h in 10 mM sodium phosphate buffer pH 7.4 at RT and shaking (600 rpm) was found to be the best condition at which the amount of oligomers residing in the middle of the gradient was maximized without the appearance of signals in bottom fractions, indicating the absence of large or fibrillar aggregates in the sample at the investigated time point. The generated oligomeric species were located in fractions 4 to 6. In this area of the gradient, proteins with *s*-values of about 7 S and molecular weights in the range of 66 to 150 kDa were expected following the centrifugation step. This expectation is based on the analysis of the distribution of standard proteins used for the calibration of the gradient (Supplementary Figure 1). The concentration of iodixanol (OptiPrep, AXIS-SHIELD, Oslo, Norway) was about 20% (w/v), giving a density of 1.112 g/cm^3^. Based on the proteins used for the calibration of the gradient, the size of oligomers in these fractions was in the range of 50 to 150 kDa.
2. Our previously described mirror image phage display selected d-peptide D3 and a derivative thereof were applied as drug candidates. Compounds were added to the pre-incubated Aβ(1–42) solution 40 min before loading the sample onto the gradient.
3. *Density gradient centrifugation*^4^,^5^,^31^ offers an approach to fractionate aggregates formed by Aβ(1–42) by their size and shape for further characterization. Analysis of solutions of synthetic Aβ(1–42) by iodixanol density gradient centrifugation revealed the *s*-value distribution of monomers and assemblies at the time point of analysis. A discontinuous gradient of iodixanol was pre-formed by layering 260 μl of 50% (w/v) iodixanol at the bottom of an 11 × 34 mm polyallomer centrifuge tube, overlaid by 260 μl of 40% (w/v), 260 μl of 30% (w/v), 780 μl of 20% (w/v), 260 μl of 10% w/v), and 100 μl of 5% (w/v) iodixanol. The total volume of the with 10 mM sodium phosphate (pH 7.4) buffered non-linear gradient was 1920 μl. The top of the gradient was overlaid by a 100 μl aliquot of incubated Aβ(1–42) in 10 mM sodium phosphate buffer pH 7.4. The samples were spun at 259,000 × *g* for 3 h at 4 °C in a TL 100 ultracentrifuge with a TLS-55 rotor (both Beckman Instruments, Brea, USA). After the centrifugation, 14 fractions of 140 μl were harvested with a pipette by upward displacement. The pellet of each tube (ca. 60 μl remaining volume) was mixed with 60 μl 6 M guanidinium hydrochloride and boiled for 10 min. The resulting solution represents fraction 15. Fraction 1 from the top of the gradient was the least dense, and fraction 15 from the bottom was the densest fraction.
4. All the fractions were analyzed with respect to their Aβ(1–42) content by RP-HPLC (see below for a detailed description).

As a control for each density centrifugation run, a sample without the added agent was used. Statistical evaluation of experimental results from eleven independent control density gradient centrifugation analyses demonstrated the good reproducibility of the procedure (Fig. 4a, black bars).

Please note: All ligands (inhibitors), but ZAβ3W, used in this work are small molecules when compared with Aβ(1–42). Therefore, even 1:1 stochiometric complexes will not increase the molecular weight of Aβ(1–42) oligomers enough to be detected as significant difference in the density gradient centrifugation.

### Analytical RP-HPLC

Quantifications of Aβ(1–42) present in DGC fractions were performed by RP-HPLC on a Zorbax SB-300 C8 column (5 μm, 4.8 × 250 mm, Agilent, Waldbronn, Germany) connected to an Agilent 1260 Infinity system. Denaturation of Aβ(1–42) assemblies and separation of Aβ(1–42) from other compounds, especially from the density gradient forming iodixanol, was achieved using 30% (v/v) acetonitrile, 0.1% (v/v) trifluoroacetic acid in H_2_O as the mobile phase, an elevated column temperature of 80 °C and a flow rate of 1 ml/min. Applied sample volumes were 20 μl. Eluting substances were detected by their UV absorbance at 215 nm. Data recording and peak area integration was achieved by the program package ChemStation (Agilent, Waldbronn, Germany). Calibration of the column was achieved with Aβ(1–42) solutions of known concentrations (0 to 25 μM Aβ(1–42)) and the resulting linear equation from a plot of peak area *vs*. Aβ(1–42) concentration allowed the calculation of molar Aβ(1–42) concentrations (from corresponding peak areas) (Supplementary Figure 9).

The quantitative analysis of Aβ(1–42) amounts in every fraction allows us to determine the recovery rate *R*:


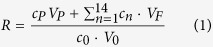


with *c*_P_ the Aβ(1–42) concentration in the pellet (15^th^ fraction), *c*_n_ the Aβ(1–42) concentrations in fractions 1 to 14, *c*_0_ the Aβ(1–42) concentration in the initial sample, *V*_f_ the volume of the fractions 1 to 14 (140 μl), *V*_P_ the volume of the pellet (15^th^ fraction), and *V*_0_ the volume of the initial sample. In the case the calculated recovery rates were higher than 100%, the Aβ(1–42) contents of all fractions were divided by the effective recovery rate.

Limiting for detection of Aβ(1–42) was the sensitivity of the UV detector connected to the HPLC. In our case the sensitivity limit was 20 nM Aβ(1–42). In principle, the sensitivity can easily be increased for example by coupling of RP-HPLC with mass spectrometry, but there was no need to do that for the experiments described in the manuscript. Thus, the QIAD could be easily performed with nano- or even picomolar Aβ concentrations.

### Animals

For study #1 23 four months old female tg/tg TBA2.1 (insertion of precursor of N-terminally modified pyroglutamate-Aβ in C57BL/6 x DBA1 background) and for study #2 19 four months old female Tg-SwDI mice (human APP with Swedish K670N/M671L, Dutch E693Q and Iowa D694N mutations on a C57BL/6 background) were used. Group sizes were decided on the basis of data from previous studies^6^. Inclusion and exclusion criteria were determined in advance. Because of the known heterogeneity of phenotypes between different sexes, only female mice with an age between 3, 5 and 4 months were included in the study. Exclusion criteria were defined as weight loss of ≥15%, surgical site infection and very serious general conditions in addition to the normal phenotype of Tg-SwDI or TBA2.1 mice. All animals fulfilled the inclusion criteria and no animal was excluded after enrolment in the study.

The animals were housed 4/cage in a controlled environment (temperature 22 °C, humidity 50–60%, light from 07:00 to 19:00), food and water were available *ad libitum*. The implantation of the Alzet minipumps was performed intraperitoneal (for study #1, model #1004; delivery rate: 0.11 μl/h duration: 4 weeks; for study #2 model #2004; delivery rate: 0.25 μl/h, duration: 4 weeks). During the treatment the animals were housed individually.

In study #1, the D3D3 peptide (*n* = 8) was compared with the D3 peptide (*n* = 8) and the control (*n* = 7, phosphate buffered saline). The applied peptide amount was 2.8 mg/pump.

In study #2, treatment took place with the D3D3 peptide (*n* = 10) and control group (*n* = 9, saline). Animals were divided into two groups based on similar average body weight. The applied peptide amount was 1 mg/pump, 0.9 mg of unconjugated peptide and 0.1 mg peptide conjugated with fluorescein.

The Alzet minipumps were filled with the appropriate solutions and implanted into the peritoneal cavity.

### Behavior and functional assessment

In study #1, selected tests from the primary screening of the SHIRPA test battery^16^ were used to assess levels of spinocerebellar function, muscle and lower motor neuron functions, sensory function, neuropsychiatric function and autonomic function of the TBA2.1 mice. The following tests were used: abnormal body carriage, alertness, abnormal gait, startle response, loss of righting reflex, touch response, pinna reflex, cornea reflex, forelimb placing reflex, hanging behavior and pain response. An arena of 55 cm × 19.5 cm × 33 cm (L × H × W) was used for individual observation and analysis. The scoring values were from 0 (similar to wild type) to 3 (extremely changed compared to wild type). Before starting the treatment, mice were stratified into treatment groups using the aforementioned tests and two days before the end of the treatment they were tested again. Experimenters were blind to group allocation. Additionally, the motor balance and motor coordination of TBA2.1 mice were determined on the rotarod for 2 days before start and end of treatment using a published protocol^15^. In the morning of the first day mice were trained to stay on the rod for at least 60 s at 10 rpm. The three following test sessions, one in the afternoon and two at the second day, started with habituation for 30 min before the mice ran three times on the rotarod with an accelerated beam from 4 till 40 rpm in 5 min. After mice falling off the rod latency time was recorded.

During the last week of treatment in study #2, animals were tested in four different behavioral tests to assess cognition and to monitor side effects. Experimenters were not informed about group allocation. First the open field test was conducted. The maze consisted of an arena of 42 cm × 42 cm with clear Plexiglas sides (20 cm high). The animal was put into the arena and observed for 4 min, with a camera driven tracker system, Ethovision 8.5 (Noldus, Wageningen, The Netherlands). The arena was subdivided into two areas, the “open” center and the area by the wall. The system recorded the position of the animal in the arena at 5 frames/second and the data were analyzed regarding time spent in each area (center *vs.* wall), similarly speed and distance were recorded. For disinfection the apparatus was wiped down with chlorhexidine and 70% ethanol and allowed to airdry.

Next, the zero maze test was conducted. The maze consisted of a circular arena with a diameter of 65 cm and four areas of equal size, two without walls, and two with walls of nontransparent material. The animal was put in the arena, and observed for 4 min, with a camera driven tracking system, Ethovision 8.5. The time spent in each area (open *vs*. closed) was recorded, similarly speed and distance were also recorded.

Subsequently the object recognition test (ORT) was carried out in a maze consisting of a rectangular polycarbonate box, with partitions separating the box into three chambers. The partitions had openings that allowed the animal to move freely from one chamber to another. The animal was monitored by the Ethovision 8.5 tracking system 8.5. On day one two objects were placed on each side of the box. The mouse was placed in the box and allowed to move freely throughout the apparatus for a 10-minute test session. After 24 h, a new object replaced one of the “old” objects and the mouse was put in the box and allowed to move freely throughout the box over a 4-minute test session. The time spent with each object and the transitions between the objects were recorded. The apparatus was wiped down with chlorhexidine followed by ethanol and water and dried with paper towels for each mouse tested.

Finally, the mice were tested for 5 days in a Morris water maze (MWM). The maze consisted of a blue circular tank of clear water (23 ± 1 °C). The mice were placed in the water at the edge of the pool and allowed to swim in order to locate a hidden, but fixed escape platform, using extra maze cues. On day 1, the mice were placed in the pool and allowed to swim freely for 60 s or until the hidden platform was found; each animal was tested for four trials per day. A maximum swim time per trial of 60 s was allowed; if the animal did not locate the platform in that time, it was placed upon it by the experimenter and left there for 10 s. The intertrial interval was 120 s. Each start position (east, north, south and west) was used equally in a pseudo random order and the animals were always placed in the water facing the wall. The platform was placed in the middle of one of the quadrants of the pool (approximately 30 cm from the side of the pool). The mouse’s task throughout the experiment was to find the platform. The animal was monitored by Ethovision 7.1.

### Statistics

Data were averaged and represented as mean ± standard error of mean (SEM). Statview, Version 5.0.1 served for all calculations, p > 0.05 was considered not significant (“n.s.”).

#### SHIRPA

Repeated measures ANOVA before v/s after *p* = 0.0027, Paired t-test (hypothesized difference ≥0) before v/s after, **p = 0.010, *p = 0.035.

#### Object Recognition Test

Paired t-test (hypothesized difference ≥0) before v/s after, *p = 0.016.

#### Plaque Load

Unpaired t-test (hypothesized difference ≥0) D3D3 v/s saline, *p = 0.020, **p < 0.0001.

#### QIAD Assay

SigmaPlot, Version 11.0 served for statistical calculations.

Paired Mann-Whitney U-test. Aβ without ligand *n* = 11, Aβ with D3, D3D3, EGCG, homotaurine, scyllo-inositol, KMS88009 and ZAβ3W *n* = 4.

*P ≤ 0.05, **P ≤ 0.01 (fraction 4: Aβ vs Aβ + D3, *p* = 0.005; Aβ + D3 vs Aβ + D3D3, *p* = 0.029;

fraction 5: Aβ vs Aβ + D3, *p* = 0.005; Aβ + D3 vs Aβ + D3D3, *p* = 0.029;

fraction 6: Aβ vs Aβ + D3, *p* = 0.007; Aβ + D3 vs Aβ + D3D3, *p* = 0.029).

The detailed descriptions of further methods like atomic force microscopy, cell culture and differentiation, cell viability assay, ThT fibril formation assay, circular dichroism spectroscopy, histopathology, synthesis of (E)-4-(2-(6-methoxybenzofuran-2-yl)vinyl)-N,N-dimethylaniline are given in the Supporting Information.

## Additional Information

**How to cite this article**: Brener, O. *et al.* QIAD assay for quantitating a compound’s efficacy in elimination of toxic Aβ oligomers. *Sci. Rep.* **5**, 13222; doi: 10.1038/srep13222 (2015).

## Supplementary Material

Supporting Information
