# Supplementary material for: QIAD assay for quantitating a compound’s efficacy in elimination of toxic Aβ oligomers
Source: Sci Rep. 2015 Sep 23;5:13222. doi: 10.1038/srep13222 (PMC4585794; doi:10.1038/srep13222)
Supplement: Supporting Information [file srep13222-s1.doc]

Supplementary Information

**QIAD assay for quantitating a compound’s efficacy in elimination of toxic Aβ oligomers**

Oleksandr Brener, Tina Dunkelmann, Lothar Gremer, Thomas van Groen, Ewa A. Mirecka, Inga Kadish, Antje Willuweit, Janine Kutzsche, Dagmar Jürgens, Filipp Oesterhelt, Stephan Rudolph, Markus Tusche, Patrick Bongen, Jörg Pietruszka, Karl-Josef Langen, Hans-Ulrich Demuth, Arnold Janssen, Wolfgang Hoyer, Susanne A. Funke, Luitgard Nagel-Steger, and Dieter Willbold*

**Table of Contents**

**1. Supporting Experimental Section**

**2. Supplementary Figures**

Supporting Experimental Section

**Peptides.** D3 (rprtrlhthrnr, all amino acids are d-enantiomers) and D3D3 (rprtrlhthrnrrprtrlhthrnr, all amino acids are d-enantiomers) with >95 % purity were purchased from JPT (Berlin, Germany) and Aβ(1-42) with 95.2 % purity from Bachem (Heidelberg, Germany).

**Previously characterized agents.** **(−)-*cis*-3,3′,4′,5,5′,7-Hexahydroxy-flavane-3-gallate** (Epigallocatechin gallate (EGCG)) with >98 % purity, 3-amino-1-propanesulfonic acid (Homotaurine) with >97 % purity and 1,3,5/2,4,6-hexahydroxycyclohexane (*scyllo*-Inosito**l)** with >98 % purity were purchased from Sigma-Aldrich ([St. Louis](http://en.wikipedia.org/wiki/St._Louis,_Missouri), [United States](http://en.wikipedia.org/wiki/United_States)).

**Preparation of Aβ peptide solutions.** In order to dissolve any pre-existing aggregates of Aβ(1-42) and to assure the monomeric state of Aβ(1−42) the sample was pre-dissolved in 1,1,1,3,3,3-hexafluoro-2-propanol (HFIP; Sigma-Aldrich, Germany) at 1.43 mg/ml and incubated over night at room temperature. HFIP was removed by evaporation for 30 min in the hood followed by a drying step in a centrifugal evaporator (RVC 2-18, Christ, Mainz, Germany) for a further 30 min. The dried Aβ was stored at −20 °C until use.

**Atomic force microscopy (AFM).** AFM was carried out for the Aβ oligomers in solution using a MFP 3D AFM (Asylum Research, Santa Barbara, USA) or for Aβ fibrils under air dried conditions using a NanoWizard II AFM (JPK Instruments AG, Berlin, Germany). Aβ(1-42) oligomers were prepared by adsorbing 40 µl of undiluted samples (pooled fractions 5 and 6) for 30 to 40 min to freshly cleaved mica followed by thorough washing with 10 mM sodium phosphate buffer, pH 7.4. The samples were imaged in the same buffer after a drifting-equilibration time of ca. 30 min in contact mode using sharp nitride levers (SNL-10) (Bruker, Camarillo, US) with an average tip radius of 7 nm and a scan rate of 1 to 2 lines per second. All images were processed using the Igor Pro 6.22A software. The images were levelled by mean plane subtraction to correct for tilting of the sample stage and servo range errors. The evaluation of the dimensions of Aβ oligomers was done by measuring the cross section of the particles perpendicular to the scanning direction. Every single cross section was fitted by a Gaussian function. The resulting radii at half-height and the fitted height are shown as histograms (Supplementary Figure 2). For statistics, we evaluated 531 particles collected from a surface area of 22 µm^2^ from up to four independent Aβ-oligomer preparations. Height and radius histograms were fitted by Gaussian functions.

The particles observed in combined DGC fractions 5 and 6 were of spherical nature. The statistical analysis for 531 particles resulted in a mean height of 4.7 nm and an average radius of 9.9 nm. In contrast to the height, the width had to be corrected for tip broadening to 8.7 nm. The calculation of the oligomer width corrected for tip artefacts was done by a routine written in MATLAB based on a tangent circle as model for the cantilever tip and an ellipse for the Aβ oligomer. Under the assumption of an oblate spheroid with an axial ratio two the particle volume was calculated to be 185 nm^3^. This represents the volume sum of the Aß(1-42) assembly and hydration water of 0.4 g/g protein. To calculate the volume of the hydration water, a density 15 % higher than bulk water was used. After subtraction of the hydration water the remaining mass corresponds to about 23 monomeric units. Based on the calculated shape and density the oligomeric species would have a sedimentation coefficient of 7.7 S, which agrees with the sedimentation coefficient deduced from the position of the particles within the density gradient.

To test for a possible correlation between radii and heights of Aß(1-42) oligomers a plot of height *vs.* radius for the measured particles was drawn (Supplementary Figure 2). In the plot no correlation between the distribution of radii and heights of Aß(1-42) oligomers can be detected, which indicates the absence of differently sized particle populations. Consequently the width of the measured distributions is primarily due to measurement errors and tip radius variation.

Samples taken from the higher DGC fractions or from fibril preparations produced for toxicity assays were checked for the presence and nature of Aß(1-42) fibrils by AFM. Following adsorption of 40 µl sample volumes for 30–40 min to freshly cleaved mica and thorough washing with ultrapure water, imaging was performed in the intermittent contact mode with silicon tips on a silicon cantilever (OMCL 160TS R3) (Olympus, Muenster, Germany) with a typical tip radius of 7 nm, a spring constant of 26 N/m, a drive frequency of 300 ± 100 kHz and a scan rate of 0.5−1 lines per second.

**Cell culture and differentiation.** SH-SY5Y cells (Leibniz Institute DSMZ-German Collection of Microorganisms and Cell Cultures) were cultured in DMEM/F-12 medium supplemented with 10 % heat-inactivated FBS, penicillin (100 U/ml), streptomycin (100 µg/ml) (all from Invitrogen, Darmstadt, Germany) in a humidified, 5 % CO_2_, 37 °C incubator and grown for a maximum of 20 passages. For the MTT cell viability assay, the cells were plated in a 96-well tissue culture plate at a density of 20,000 cells/well and 24 h after seeding the cells were treated with all-*trans* retinoic acid (RA) (Sigma-Aldrich, Taufkirchen, Germany) at a final concentration of 10 μM in a culture medium containing 1 % FBS. After 6 days of RA treatment, the cells were grown for a further 2 days in a serum-free medium containing human recombinant BDNF (Sigma-Aldrich, Taufkirchen, Germany) at a final concentration of 2 nM.

PC12 cells (Leibniz Institute DSMZ-German Collection of Microorganisms and Cell Cultures) were cultured in DMEM/F-12 medium supplemented with 10 % heat-inactivated FCS, 5 % HS, penicillin (100 U/ml), streptomycin (100 µg/ml) (all from Invitrogen, Darmstadt, Germany) in a humidified, 5 % CO_2_, 37 °C incubator and grown for a maximum of 12 passages. For the MTT cell viability assay, the cells were plated in a 96-well tissue culture plate at a density of 10,000 cells/well and 24 h after seeding the cells were treated with Aβ(1-42) solutions with or without an agent.

**MTT cell viability assay.** The toxicity of Aß(1-42) oligomers was assessed using the MTT assay in differentiated SH-SY5Y cells. Aß(1-42) oligomers pooled from DGC fractions 5 and 6 were added to cells at a corresponding monomer concentration of 4, 2 and 0.8 µM, as determined by RP-HPLC. The concentration of iodixanol in the controls without Aß(1-42) was adjusted to match that present in oligomer fractions 5 and 6. The data were not normalized to the value of the untreated control, because the toxic effects of Aß(1-42) oligomers and iodixanol might not be additive. Following 24 h, MTT at a final concentration of 0.5 mg/ml was added to the cells for 4 h at 37 °C. Finally, formazan salt crystals were solubilized using 10 % SDS and 0.01 M HCl, and the absorbance was measured at 565 nm in an Infinite M1000 plate reader (Tecan, Maennedorf, Switzerland) (Fig. 3c and Supplementary Figure 11).

We tested the toxicity of our DGC prepared oligomers on RA/BDNF differentiated neuroblastoma cells, which have been shown to be susceptible to Aß(1-42) oligomers. These Aß(1-42) oligomers were proven to be highly toxic to RA/BDNF differentiated cells and were less toxic to undifferentiated cells. The difference of toxicity between differentiated and undifferentiated cells was not statistically quantified.

The bulk toxicity of Aβ(1-42) and the inhibitory effects of D3 and D3D3 were assessed using the MTT assay in PC12 cells. Aβ(1-42) was pre-incubated to enrich the amount of Aβ oligomers in the same way as for the QIAD assay (80 µM Aβ(1-42), 4.5 h in 10 mM sodium phosphate buffer pH 7.4 at RT and shaking). Subsequently either 16, 80 or 400 µM D3 or D3D3 were added to the Aβ solution and coincubated for further 40 min. Aβ solution with or without agent were applied to PC12 cells with resulting final Aβ concentration of 1 µM and 0.2, 1 or 5 µM D3 or D3D3. The toxicity of Aβ with or without agent was assessed using the MTT assay (as described above). The data were normalized to the value of the untreated control (Supplementary Figure 11).

**ThT fibril formation assay**. 1 or 10 µM solutions of D3 or D3D3 were prepared in 1 x PBS pH 7.4 with 10 µM Thioflavin T. The solutions were each added to Aβ(1-42) films to gain a final Aβ concentration of 10 µM Aβ(1-42) in the approach. 50 µl of each experimental approach were pipetted in a black 384-well plate (Greiner 384 Well polypropylene Greiner Bio-One GmbH, Frickenhausen, Germany) in septuplicate. An approach with 10 µM Aβ(1-42) without addition of peptide served as positive control, 10 µM ThT in 1 x PBS and 10 µM D3 or D3D3, respectively, served as negative controls. The relative fluorescence was measured every 30 min for >24 h at a POLARstar OPTIMA plate reader (BMG Labtech GmbH, Ortenberg, Germany) at 25 °C (excitation at 440 nm; emission at 490 nm). Prior to each measurement, the plate was shaken for 60 s in double orbital shaking mode. At the point in time when 10 µM Aβ(1-42) reached saturation, the relative fluorescence values of 10 µM Aβ(1-42) were normalized to 100% and values of the other experimental approaches were set in correlation to this value. The experiment was repeated eight times and the average of the obtained values as well as the standard deviation was calculated (Supplementary Figure 10).

**Circular dichroism (CD) spectroscopy.** The high UV absorbance of the gradient forming iodixanol prohibited a direct CD spectroscopic analysis of oligomeric Aß(1-42) preparations derived from DGC. Therefore, immediately before CD measurements iodixanol was removed from freshly prepared Aß(1-42)-oligomer preparations by SEC on two 5 ml Sephadex G-25 columns in series equilibrated in 10 mM sodium phosphate (pH 7.4) (HiTrap Desalting, GE Healthcare, Freiburg, Germany) on an ÄKTA purifier system (GE Healthcare, Freiburg, Germany). Typically, 240 to 480 µl of 17.3 µM Aβ oligomers (expressed as molar concentration of Aβ monomers) were applied to SEC. Eluting compounds were monitored by their UV absorbance at 215 nm and collected in 0.4 ml fractions. The Aß(1-42) oligomers eluted in the void volume. The concentration was measured by RP-HPLC as described above and is given as molar concentration of Aß(1-42) monomers. Typical yields were in the range of 5 to 10 µM and sufficient for CD measurements. Far-UV CD spectra of Aß(1-42) oligomers were recorded on a JASCO J-815 instrument at 20 °C using a quartz cuvette with 1 mm path length and instrument settings as follows: 0.2 nm step size, 20 nm min^-1^ scan speed, and 1 nm band width. The signal-to-noise ratio was improved by accumulation of 10 scans per sample. Resulting CD spectra were corrected by subtraction of the corresponding buffer spectra (Fig. 3b). The mean residue ellipticity
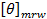
 in deg·cm^2^·dmol^-1^ was calculated from the equation
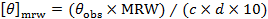
, where
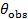
, observed ellipticity (in degrees);
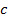
, concentration (in g/ml);
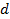
, cell path length (in cm);
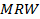
 (mean residue weight), molecular weight divided by number of peptide bonds.

**Histopathology.** Four weeks after the implantations, the animals were sacrificed for histopathological analysis. Therefore, the mice were anesthetized and transcardially perfused. The brains were then removed and the right hemisphere was fixed overnight in 4 % paraformaldehyde and cryoprotected in 30 % sucrose for 24 h followed by antifreeze in 15 % sucrose and 30 % ethylene glycol in 0.05 M phosphate buffer, pH 7.4. Subsequent six series (1 in 6) of coronal sections (30 μm) were cut through the brain. The first series of sections was mounted unstained; the second and third series were stained immunohistochemically, for human Aβ using the W0-2 antibody, for inflammation using mouse anti-GFAP (Sigma) and rabbit anti-mouse Iba1 (WAKO), according to published protocols.[^1^](#_ENREF_1) The other series were stored in antifreeze at −20 °C.

**Quantification of Aβ plaque load in hippocampus and cortex.** The appropriate areas (dorsal hippocampus and frontal cortex) of the brain were digitized using a Olympus DP70 digital camera.[^2^](#_ENREF_2) To avoid changes in lighting, which might affect measurements, all images were acquired in one session. Further, to avoid differences in staining density between sections, the measurements were performed on sections that were stained simultaneously, i.e., in the same staining tray (n=24). The percentage of area covered by the reaction product to Aβ was measured [^1^](#_ENREF_1) in the hippocampus and midline, frontal cortex using the ScionImage (NIH) program.[^2^](#_ENREF_2) Using a similar procedure, using digital images to be able to overlay the defined measurement area, plaques were counted in the same brain area on the adjacent sections that were stained with Congo red.  The density of GFAP or CD11b staining was measured by placing a standard sized circle (200 µm diameter) around the plaque core (stained with Congo red), and measuring the optical density of the staining in the circle using the ScionImage (NIH) program. All density measurements were done in triplicate, i.e., measuring the standardized area around three plaques at three different levels of the dorsal hippocampus and the frontal, midline cortex (**Supplementary Figure 5**). These measurements were done in triplicate in sections (that had been stained simultaneously) by an observer blinded to the treatment of the animal.[^2^](#_ENREF_2) Data were analyzed by ANOVA (Systat 11; between groups), and post-hoc tests (Tukey and Scheffe) were carried out to determine the source of a significant main effect or interaction.

**Synthesis of (*E*)-4-(2-(6-methoxybenzofuran-2-yl)vinyl)-*N*,*N*-dimethylaniline (4)** (**Supplementary Figure 8**)

(according to the literature [^3^](#_ENREF_3)).

*Synthesis of ethyl 6-methoxybenzofuran-2-carboxylate* (**1**): To a solution of 2-hydroxy-4-methoxybenzaldehyde (4.56 g, 30 mmol) in dry DMF (17 ml) potassium carbonate (8.30 g, 60 mmol) was added. Ethyl bromoacetate (3.43 ml, 30 mmol) in DMF (15 ml) was added at r.t. and under inert conditions. The reaction mixture was stirred for 6h at 130 °C. The mixture was diluted with water (150 ml) and extracted with CH_2_Cl_2_ (100 ml), dried over MgSO_4_ and concentrated under reduced pressure. Flash chromatography afforded the product as white solid (3.73 g, 16.9 mmol, 56%). ^1^H-NMR (600 MHz, CDCl_3_) δ 7.53 (d, ^3^*J*(H,H) = 8.6 Hz, 1H, arom.-CH), 7.46 (s, 1H, 3-H), 7.06 (d, ^4^*J*(H,H) = 2.0 Hz, 1H, arom.-CH), 6.94 (dd, *^4^J*(H,H) = 2.1 Hz, ^3^*J*(H,H) = 8.7 Hz, 1H, arom.-CH), 4.43 (q, ^3^*J*(H,H) = 7.1 Hz, 2H, CH_2_), 3.68 (s, 3H, OCH_3_), 1.42 (t, ^3^*J*(H,H) = 7.1 Hz, 3H, CH_3_) ppm. ^13^C-NMR (151 MHz, CDCl_3_): δ 160.5, 159.6, 157.1, 145.0, 123.0, 120.3, 114.1, 114.0, 95.8, 61.3, 55.7, 14.4 ppm. m.p.: 88.7 °C. HR-MS (ESI, cation): calculated: [C_12_H_12_O_4_+Na^+^]: *m/z*= 243.06278, found: [C_12_H_12_O_4_+Na^+^]: *m/z*= 243.06280.

*Synthesis of (6-methoxybenzofuran-2-yl)methanol* (**2**): To a solution of ethyl 6-methoxybenzofuran-2-carboxylate (**1**) (2.00 g, 9.1 mmol) in dry THF (30 ml) LiAlH_4_ (0.69 g, 18.2 mmol) was added under inert conditions. The reaction mixture was stirred at r.t. for 30 min. After full conversion surplus LiAlH_4_ was neutralized by adding small amounts of water. Resulting solid was solved by adding aqueous HCl solution followed by extraction with EtOAc (600 ml). Collected organic layer was dried over MgSO_4_, concentrated under reduced pressure and purified by flash chromatography to afford the product **2** as colorless oil (1.51 g, 8.47 mmol, 93 %). ^1^H-NMR (600 MHz, CDCl_3_) δ 7.38 (d, ^3^*J*(H,H) = 8.5 Hz, 1H, arom.-CH), 6.98 (d, ^4^*J*(H,H) = 2.2 Hz, 1H, arom.-CH), 6.85 (dd, ^4^*J*(H,H) = 2.2 Hz, ^3^*J*(H,H) = 8.5 Hz, 1H, arom.-CH), 6.55 (s, 1H, 3-H), 4.70, (s, 2H, CH_2_), 3.82 (s, 3H, OCH_3_) ppm. ^13^C-NMR (151 MHz, CDCl_3_): δ 158.0, 156.1, 155.6, 121.4, 121.2, 111.8, 104.1, 96.0, 58.0, 55.7 ppm. HR-MS (ESI, cation): calculated: [C_10_H_10_O_3_+Na^+^]: *m/z*= 201.05222, found: [C_10_H_10_O_3_+Na^+^]: *m/z*= 201.05205.

*Synthesis of diethyl ((6-methoxybenzofuran-2-yl)methyl)phosphonate* (**3**): To a solution of (6-methoxybenzofuran-2-yl)methanol (**2**) (933 mg, 5.24 mmol) in diethylether (60 ml) PBr_3_ (1.04 ml, 10.9 mmol) was added under inert conditions at 0 °C. The reaction mixture was stirred for 10 min. at 0 °C and then allowed to warm up to r.t. and stirred for another hour. Afterwards the reaction mixture was poured onto ice cooled water and extracted with diethylether (30 ml). The solvent was removed under reduced pressure to give the crude product as colorless oil. The product was filled into a round bottom flask and diluted with triethyl phosphite (10 ml). The reaction mixture was heated to 140 °C for 4 h. Afterwards triethyl phosphite was removed by vacuum distillation. The remaining crude product was purified by flash column chromatography to give the phosphonate **3** as colorless oil (1.36 g, 4.56 mmol, 87 % over two steps).^1^H-NMR (600 MHz, CDCl_3_) δ 7.36 (d, ^3^*J*(H,H) = 8.6 Hz, 1H, arom.-CH), 6.99 (d, ^4^*J*(H,H) = 2.2 Hz, 1H, 3-H), 6.84 (dd, ^4^*J*(H,H) = 2.2 Hz, ^3^*J*(H,H) = 8.5 Hz, 1H, arom.-CH), 6.56 (d, ^3^*J*(H,H) = 3.9 Hz, 1H, arom.-CH), 4.12 (m, 4H, CH_2_), 3.34 (d, ^3^*J*(H,P) = 21.0 Hz, 1H, CH_2_), 1.30 (t, ^3^*J*(H,H) = 7.1 Hz, 6H, CH_3_) ppm. ^13^C-NMR (151 MHz, CDCl_3_): δ 157.7, 155.8, 147.8, 122.0, 120.7, 111.6, 105.0, 104.9, 95.9, 62.5, 55.7, 27.7, 26.7, 16.4 ppm. ^31^P-NMR (243 MHz, CDCl_3_): δ 22.6 ppm. HR-MS (ESI, cation): calculated: [C_14_H_19_O_5_P+H^+^]: *m/z*= 299.10429, found: [C_14_H_19_O_5_P+H^+^]: *m/z*= 299.10428, calculated: [C_14_H_19_O_5_P+Na^+^]: *m/z*= 321.08623, found: [C_14_H_19_O_5_P+Na^+^]: *m/z*= 321.08620.

*Synthesis of (E)-4-(2-(6-methoxybenzofuran-2-yl)vinyl)-N,N-dimethylaniline* (**4**): To a solution of diethyl ((6-methoxybenzofuran-2-yl)methyl)phosphonate (**3**) (1.00 g, 3.35 mmol) in THF (30 ml) at 0 °C NaHMDS (738 mg, 4.02 mmol) was added. The reaction mixture was stirred for 10 min. at 0 °C and then 4-(dimethylamino)benzaldehyde (550 mg, 3.69 mmol) in THF (10 ml) was added. The reaction mixture was allowed to warm up to r.t. The mixture was stirred for another hour at r.t. Afterwards the mixture was poured onto ice cooled water and extracted with CH_2_Cl_2_ (300 ml). The solvent was removed under reduced pressure. Flash chromatography afforded the product as a yellow solid (664 mg, 2.26 mmol, 67 %). ^1^H-NMR (600 MHz, CDCl_3_) δ 7.40 (d, ^3^*J*(H,H) = 8.8 Hz, 2H, arom.-CH), 7.35 (d, ^3^*J*(H,H) = 8.4 Hz, 1H, arom.-CH), 7.16 (d, ^3^*J*(H,H) = 16.2 Hz, 1H, CH), 6.82 (d, ^4^*J*(H,H) = 2.0 Hz, 1H, arom.-CH), 6.82 (dd, ^4^*J*(H,H) = 2.2 Hz,  ^3^*J*(H,H) = 8.5 Hz, 1H, arom.-CH), 6.76 (d, ^3^*J*(H,H) = 16.1 Hz, 1H, CH), 6.71 (d ^3^*J*(H,H) = 8.6 Hz, 2H, arom.-CH), 6.48 (s, 1H, 3-H), 3.85 (s, 3H, OCH_3_), 2.98 (s, 6H, CH_3_) ppm. ^13^C-NMR (151 MHz, CDCl_3_): δ 157.9, 155.8, 155.5, 150.3, 129.3, 127.8, 125.1, 122.9, 120.5, 112.4, 112.3, 111.4, 103.1, 95.8, 55.8, 40.4 ppm. m.p.: 167 °C. HR-MS (ESI, cation): calculated: [C_19_H_20_O_2_+Na^+^]: *m/z*= 294.14886, found: [C_19_H_20_O_2_+Na^+^]: *m/z*= 294.14888.

**Supplementary Figures**

**
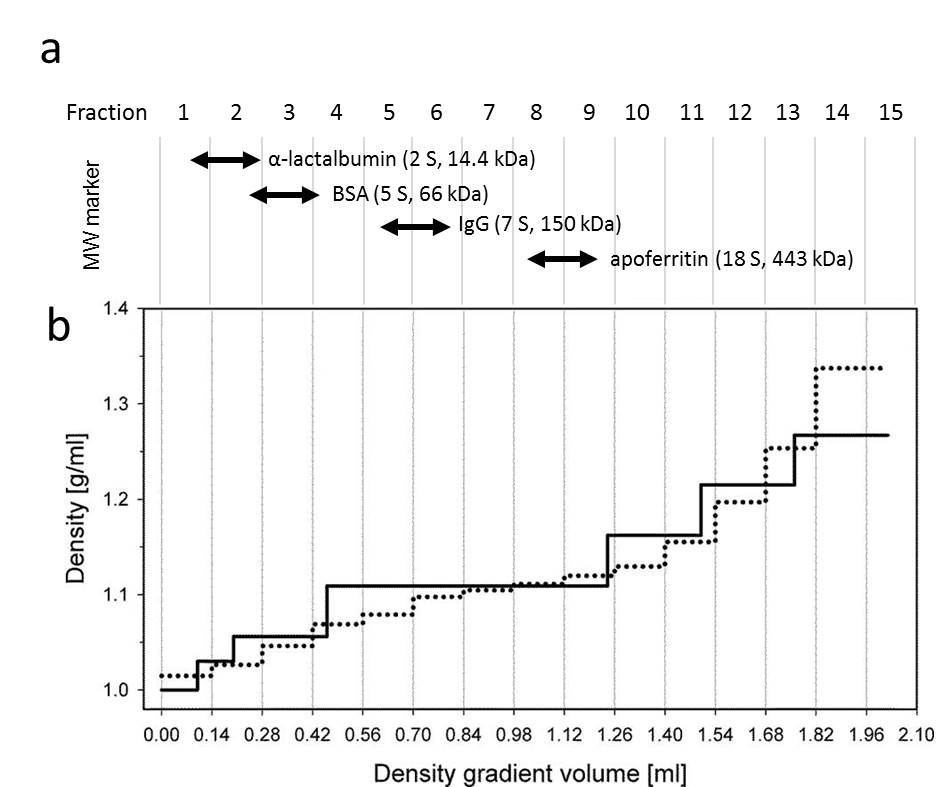
**

**Supplementary Figure 1.** Characterization of the density gradient (DG). (**a**) Position of calibration proteins (α-lactalbumin, BSA, IgG and apoferritin) in the DG after centrifugation. Due to known s-values of the calibration proteins one can estimate the size of Aβ aggregates in a certain fraction. (**b**) Refractometric density determination of DG fractions before (solid line) and after (dotted line) centrifugation. The last fraction contains the potential pellet with around 60 µl of remaining liquid volume.


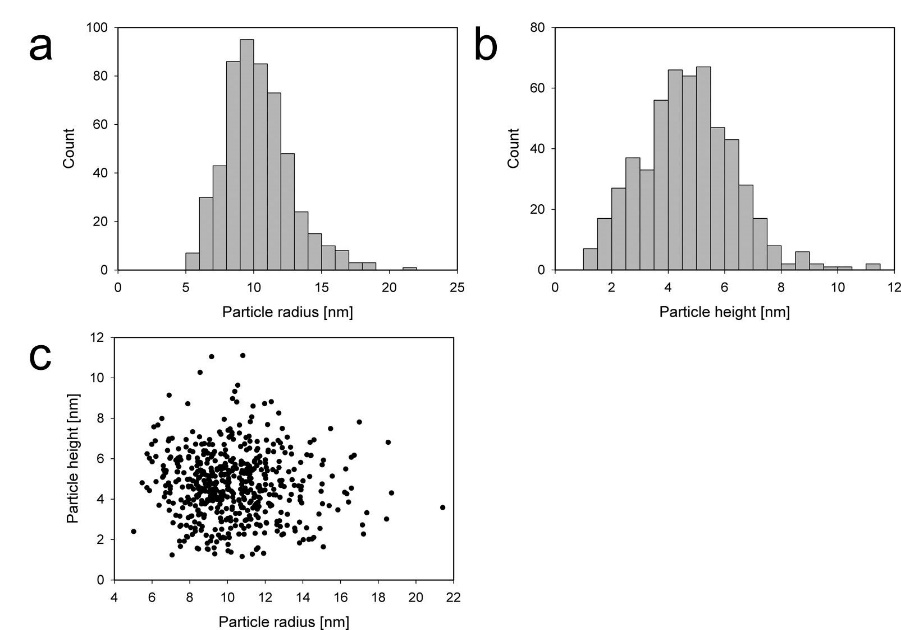


**Supplementary Figure 2.** Statistical analysis of Aβ oligomer AFM analysis (pooled fractions 5 and 6) dimensions. The AFM measurements were carried out in solvent (10 mM sodium phosphate buffer pH 7.4) in contact mode. Cross sections of Aß(1-42) oligomers (531 particles collected from 22 µm^2^ surface) perpendicular to the scanning direction were fitted by Gaussian functions. The resulting radius (horizontal dimension) at half-height (**a**) and the fitted height (vertical dimension) (**b**) are shown as histograms. By global fitting of the histograms with Gaussian functions an average height of 4.7 nm and an average radius of 9.3 nm (not corrected for the tip dimensions) were determined. (**c**) A plot of measured radii vs. heights of Aß(1-42) oligomers. No correlation between the distribution of radii and heights of measured particles can be detected indicating that the variance of different measured values is due to measurement inaccuracy and not to differently sized particle populations.

**
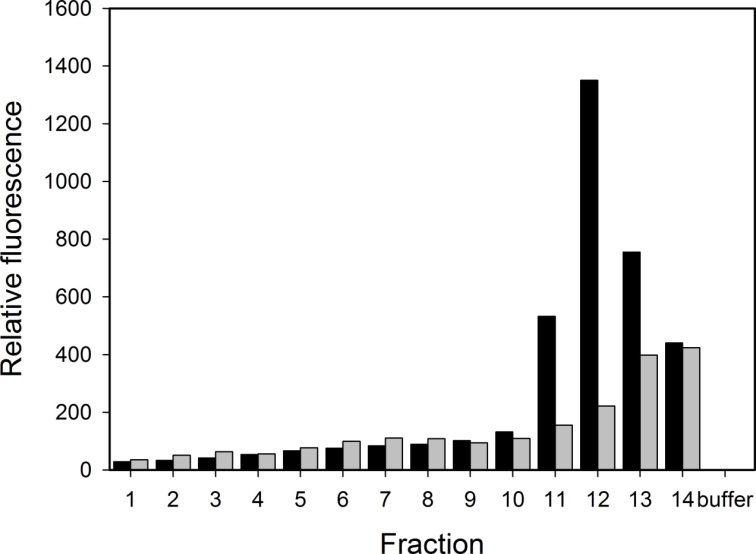
**

**Supplementary Figure 3.** Thioflavin T (ThT) test of Aß(1-42) species fractionated by DGC. ThT fluorescence was measured in freshly prepared Aß(1-42) fractions (black) and in fractions from a density gradient run without Aß(1-42) as Iodixanol control (gray) after addition of ThT. Sown is the mean of two measurements. Iodixanol alone shows a concentration dependent effect on ThT fluorescence. Only fibrillar Aß(1-42) species in fraction 11-13 show amyloid specific ThT fluorescence, while oligomer fractions did not, indicating that the secondary structure of prepared Aß(1-42) oligomers differs from the fibril’s one.


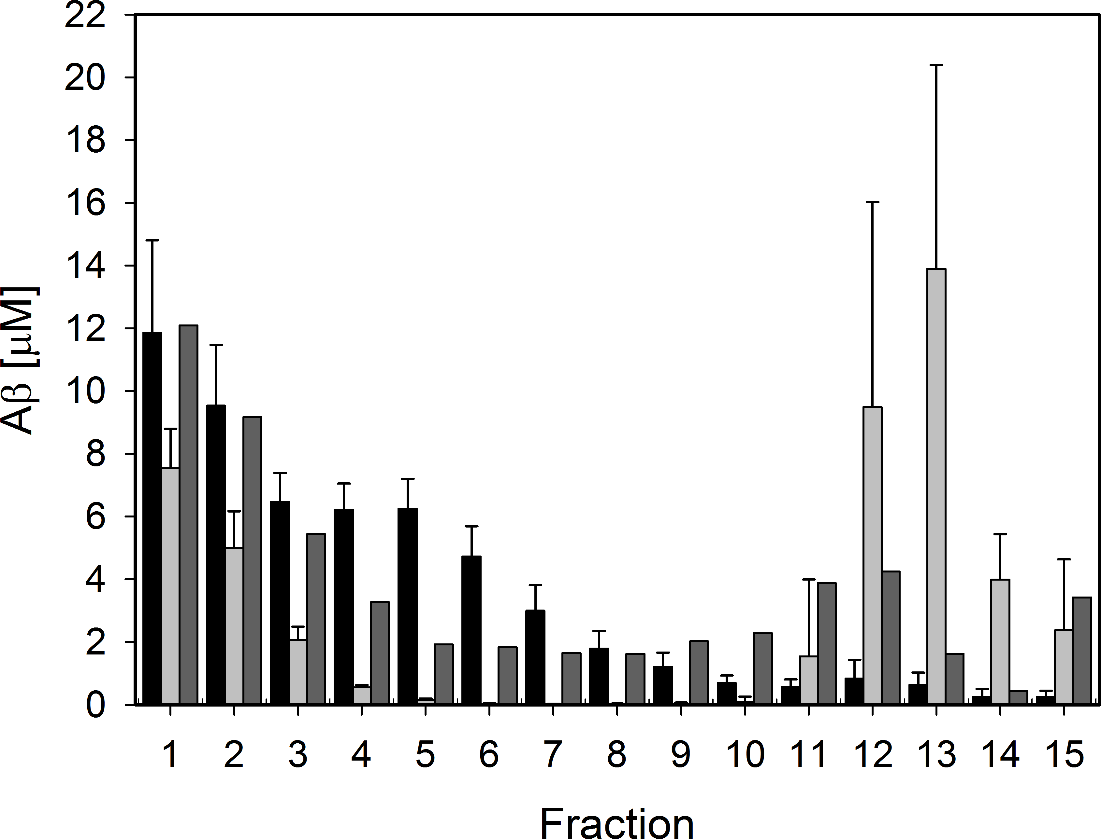


**Supplementary Figure 4.** QIAD assay. After pre-incubation of 80 µM Aß(1-42) for 4.5 h at RT and further incubation for 40 min with or without agent, Aß(1-42) size distributions in the absence (black) or presence of either 10 µM D3D3 (light gray) or 5 µM D3D3 (dark gray) were analyzed by DGC. Aß(1-42) concentrations were determined by UV absorption during RP-HPLC.


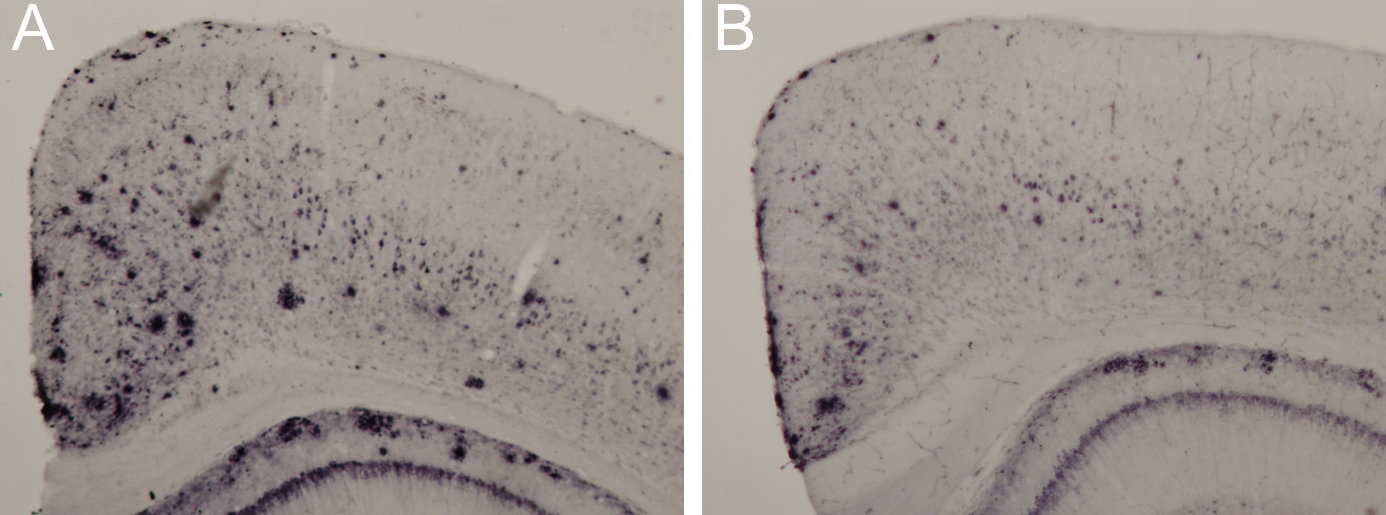


**Supplementary Figure 5.** Aß plaque load in the dorsal hippocampus and frontal cortex of Tg-SwDI mice by W0-2 antibody staining. Saline-treated (A) control compared to D3D3 treated (B).


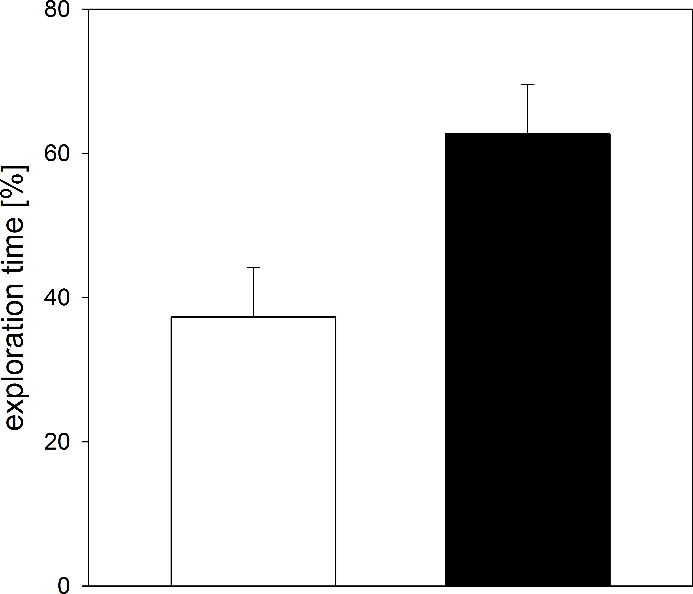


**Supplementary Figure 6.** Object recognition test of non-Tg C57BL/6 mice. Preference for the new object (black) is expressed as exploration time in comparison with exploration of a familiar object (white).


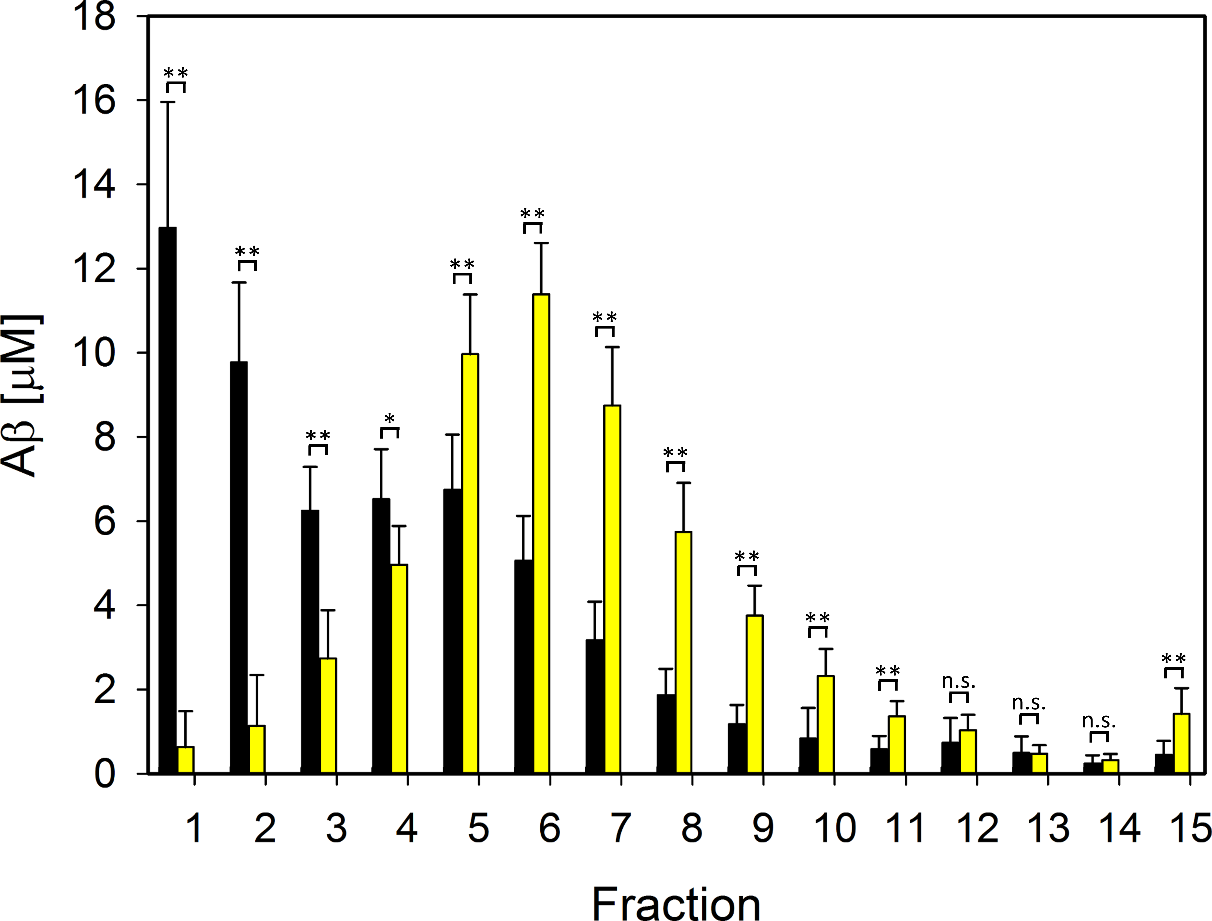


**Supplementary Figure 7.** Statistical analysis for the significance of epigallocatechin gallate (EGCG) influence on Aβ particle size distribution revealed by QIAD assay. After pre-incubation of 80 µM Aß(1-42) for 4.5 h at RT and further incubation for 40 min with or without agent, Aß(1-42) size distributions in the absence (black) or presence of 2 mM EGCG (yellow) were analyzed by density gradient centrifugation. Aß(1-42) concentrations were determined by UV absorption during RP-HPLC. Paired u-test; **p* ≤ 0.05, **p ≤ 0.01.

**Supplementary Figure 8.** Synthesis route of (*E*)-4-(2-(6-methoxybenzofuran-2-yl)vinyl)-*N*,*N*-dimethylaniline. Experimental conditions: a) K_2_CO_3_, DMF, 130 °C, 6 h, 56 %; b) LiAlH_4_, 0 °C, 2 h, 93 %; c) PB_3_, Et_2_O, 0 °C – r.t., 1 h; d) P(OEt)_3_, 140 °C, 4 h, 87 % over two steps; e) 4-(dimethylamino)benzaldehyde, NaHMDS, THF, 0 °C – r.t., 1 h, 67 %.

**Supplementary Figure 9.** Calibration of the analytical RP-HPLC column for quantitative analysis of Aß(1-42) content in density gradient fractions. Aß(1-42) solutions of known concentrations (0 to 20 µM) were analyzed by RP-HPLC. Absorbance was recorded at 215 nm. Obtained peak areas were plotted against original Aß(1-42) concentrations and fitted by a linear equation.


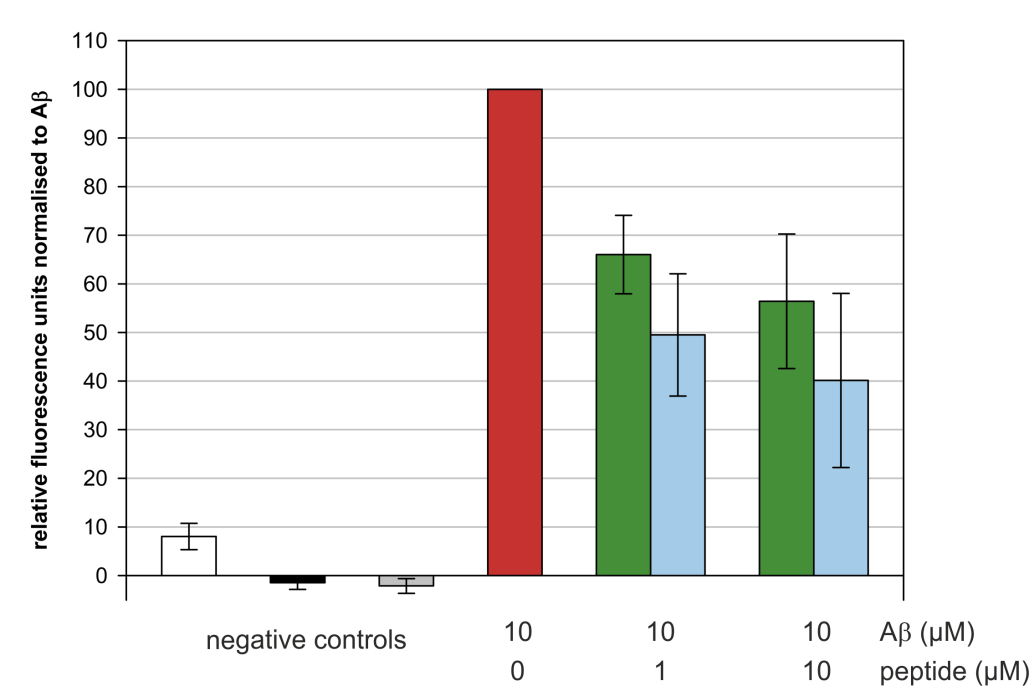


**Supplementary Figure 10.** ThT fibril formation assay: ThT fluorescence was determined after 24 h incubation of 10 µM Aß(1-42) without agents (positive control, set to 100 % fluorescence, red bar) or with 1 or 10 µM D3 (green) or D3D3 (light blue). Negative controls are 10 µM ThT (white); 10 µM D3 (black) und 10 µM D3D3 (grey).


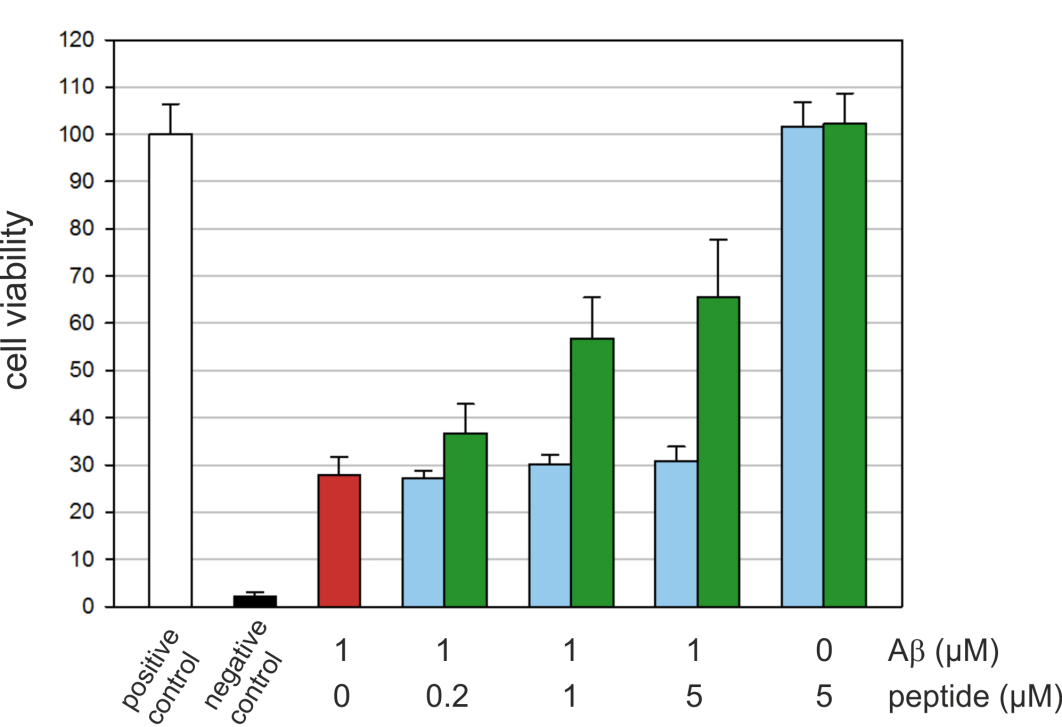


**Supplementary Figure 11.** MTT cytotoxicty assay: PC12 cells were treated with solvent buffer (positive control, set to 100 % cell viability, white bar), with 0.125 % Triton X-100 (negative control, black bar) or with combinations of none or 1 µM Aß(1-42) and 0,2, 1, or 5 µM D3 (light blue) or D3D3 (green). Cell viability was measured as absorbace at 565 nm of formed dye from MTT and normalized to 100 % viability of untreated cells. Whereas D3D3 did show a dose dependent effect on Aß toxicity, D3 did not show any cytotoxicity inhibition up to 5 µM.


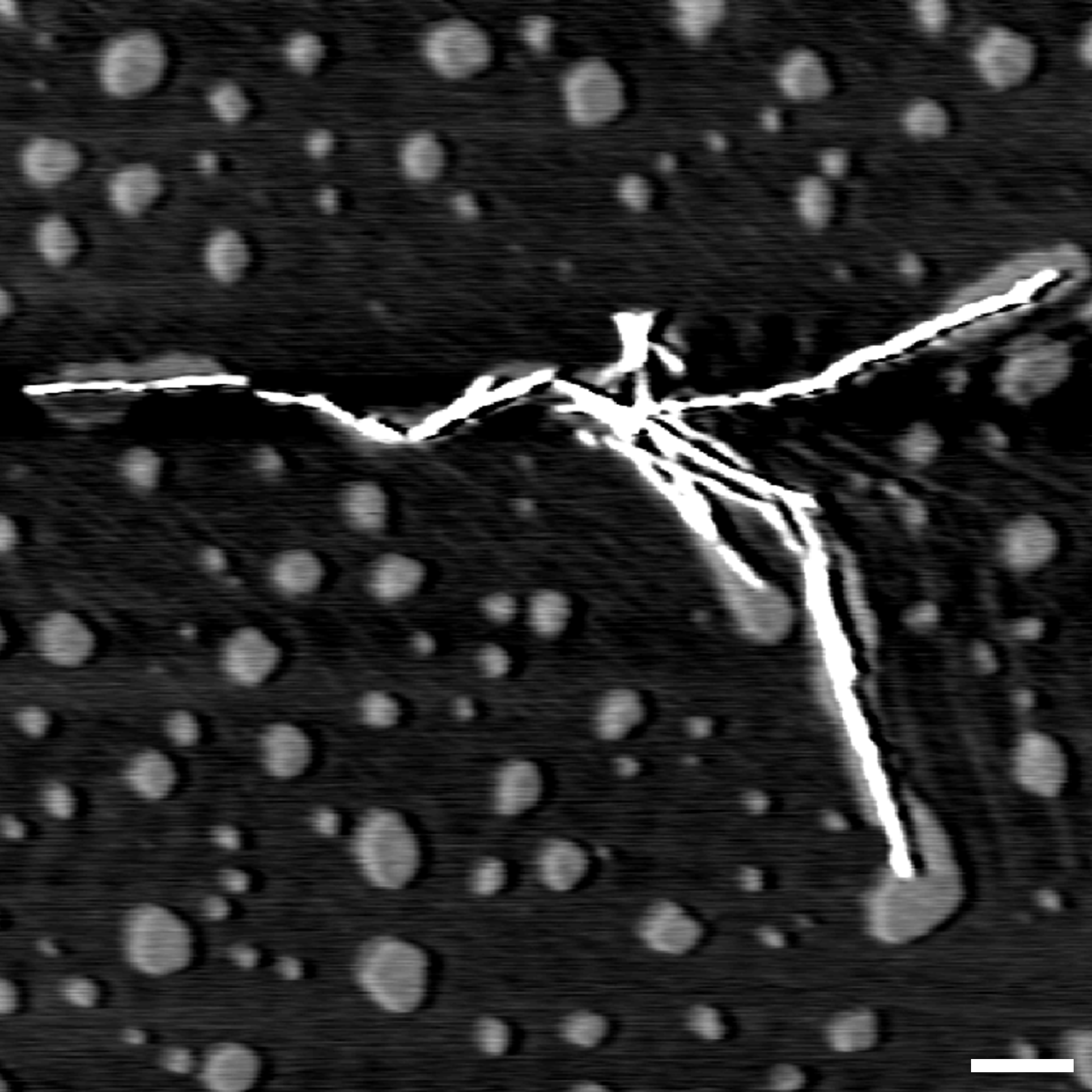


**Supplementary Figure 12.** AFM analysis of Aβ(1-42) fibrils/ aggregates from DGC fraction 12. Scale bars 200 nm**.**

| **Agent** | **Concentration** | **Stoichiometry (Aß(1-42) : agent)** |
| --- | --- | --- |
| D3 | 20 µM | 4 : 1 |
| D3D3 | 10 µM | 8 : 1 |
| Homotaurine | 2 mM | 1 : 25 |
| Scyllo-inositol | 2 mM | 1 : 25 |
| Epigallocatechin gallate | 2 mM | 1 : 25 |
| 6-methoxy-2-(4-dimethylaminostyryl) benzofuran | 200 µM | 1 : 2.5 |
| ZAß3W | 54 µM | 1.5 : 1 |

**Supplementary Table 1.** Aß(1-42) to ligands stoichiometry used in this study and displayed in main figures 2 and 3. Aß(1-42) was used at 80 µM concentration.

1 van Groen, T., Kiliaan, A. J. & Kadish, I. Deposition of mouse amyloid beta in human APP/PS1 double and single AD model transgenic mice. *Neurobiology of disease* **23**, 653-662, doi:10.1016/j.nbd.2006.05.010 (2006).

2 Kadish, I., Pradier, L. & van Groen, T. Transgenic mice expressing the human presenilin 1 gene demonstrate enhanced hippocampal reorganization following entorhinal cortex lesions. *Brain research bulletin* **57**, 587-594 (2002).

3 Byun, J. H. *et al.* Aminostyrylbenzofuran derivatives as potent inhibitors for Abeta fibril formation. *Bioorganic & medicinal chemistry letters* **18**, 5591-5593, doi:10.1016/j.bmcl.2008.08.111 (2008).
